# *catena*-Poly[[(1,3-dimethyl­imidazolin-2-one-κ*N*)(1,10-phenanthroline-κ^2^ *N*,*N*′)copper(II)]-μ-furan-2,5-dicarboxyl­ato-κ^2^ *O* ^2^:*O* ^5^]

**DOI:** 10.1107/S160053681203111X

**Published:** 2012-08-01

**Authors:** Ya-Feng Li, Yue Xu, Xiao-Lin Qin, Yong-Peng Yuan, Wen-Yuan Gao

**Affiliations:** a School of Chemical Engineering, Changchun University of Technology, Changchun 130012, People’s Republic of China

## Abstract

The polymeric title compound, [Cu(C_6_H_2_O_5_)(C_12_H_8_N_2_)(C_5_H_10_N_2_O)]_*n*_, is composed of an infinite chain formed along [100] by linking the (1,3-dimethyl­imidazolin-2-one)(1,10-phenanthroline)copper(II) units with two O atoms of two carboxyl­ate groups of the furan-2,5-dicarboxyl­ate ligand. The Cu^II^ atom, which lies on a twofold rotation axis, displays a square-pyramidal coordination. The dihedral angles of the 1,10-phenanthroline ligand with respect to the furan rings of the carboxyl­ate anions that are connected to the metal atom are 62.18 (11) and 88.27 (12)°.

## Related literature

For related structures, see: Li, *et al.* (2012*a* ▶,*b* ▶).
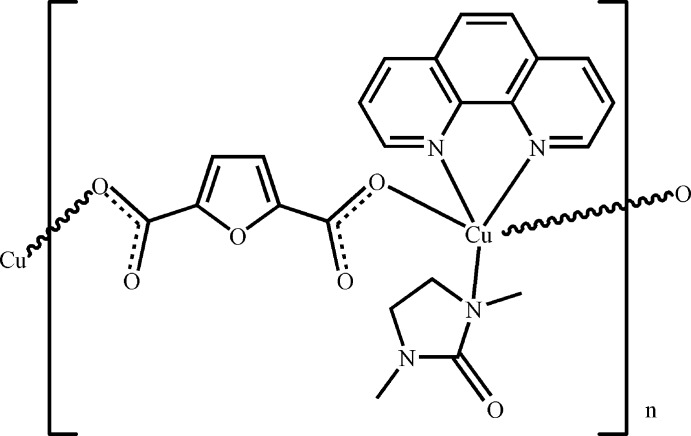


## Experimental

### 

#### Crystal data

[Cu(C_6_H_2_O_5_)(C_12_H_8_N_2_)(C_5_H_10_N_2_O)]
*M* *_r_* = 511.98
Orthorhombic, 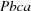

*a* = 15.620 (3) Å
*b* = 14.598 (3) Å
*c* = 18.616 (4) Å
*V* = 4244.8 (15) Å^3^
*Z* = 8
Mo *K*α radiation
μ = 1.08 mm^−1^
*T* = 293 K
0.10 × 0.10 × 0.10 mm

#### Data collection

Rigaku R-AXIS RAPID diffractometer
Absorption correction: multi-scan (*ABSCOR*; Higashi, 1995 ▶) *T*_min_ = 0.900, *T*_max_ = 0.900
30193 measured reflections
3727 independent reflections
2376 reflections with *I* > 2σ(*I*)
*R*_int_ = 0.110

#### Refinement

*R*[*F* ^2^ > 2σ(*F* ^2^)] = 0.047
*wR*(*F* ^2^) = 0.109
*S* = 1.04
3727 reflections
309 parameters
H-atom parameters constrained
Δρ_max_ = 0.46 e Å^−3^
Δρ_min_ = −0.37 e Å^−3^

### 

Data collection: *PROCESS-AUTO* (Rigaku, 1998 ▶); cell refinement: *PROCESS-AUTO*; data reduction: *CrystalStructure* (Rigaku/MSC, 2002 ▶); program(s) used to solve structure: *SHELXS97* (Sheldrick, 2008 ▶); program(s) used to refine structure: *SHELXL97* (Sheldrick, 2008 ▶); molecular graphics: *DIAMOND* (Brandenburg, 2000 ▶); software used to prepare material for publication: *SHELXL97*.

## Supplementary Material

Crystal structure: contains datablock(s) I, global. DOI: 10.1107/S160053681203111X/ng5281sup1.cif

Structure factors: contains datablock(s) I. DOI: 10.1107/S160053681203111X/ng5281Isup2.hkl

Additional supplementary materials:  crystallographic information; 3D view; checkCIF report

## Figures and Tables

**Fig. 1.**
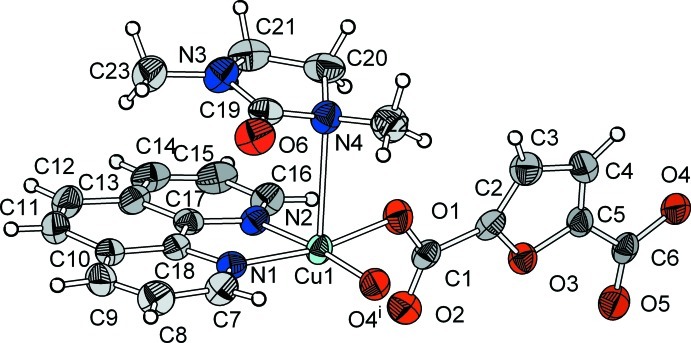
The asymmetric unit of (I), showing the atomic labelling scheme and displacement ellipsoids at the 50% probability level. [Symmetry codes: (i) 0.5 + x, y, 0.5 - z.]

**Fig. 2.**
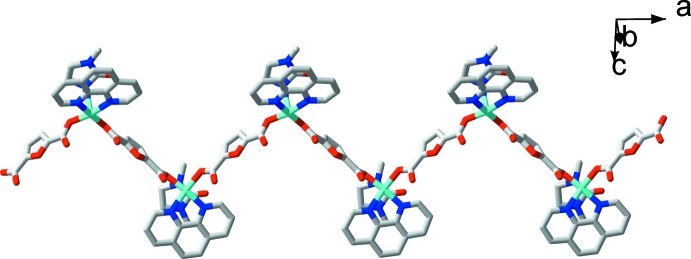
The stick plot of (I), displaying the infinite chain formed by linking the Cu with two oxygen atoms of two carboxyls of furan-2,5-dicarboxylate.

## supplementary crystallographic information

### Comment

Recently, we utilized furan-2,5-dicarboxylate anion as the ligand to synthesize MOFs (Li *et al.*, 2012*a*,*b*). In this work, we report a chainlike compound, [Cu(C_5_H_10_N_2_O)(C_12_H_8_N_2_)(C_6_H_2_O_5_)]_n_ (Scheme I), is structurally determined.

The asymmetric unit of (I) is consists of one Cu(II) cation, one furan-2,5-dicarboxylate anion, one 1,3-dimethyl-2-imidazolinone molecule and one 1,10-phenanthroline molecule (Fig.1). The Cu atom is coordinated by two carboxylate O atoms, two N atoms of one C_12_H_8_N_2_ and one nitrogen of from C_5_H_10_N_2_O. The geometry is a square pyramid. The furan-2,5-dicarboxylate shows a µ_2_:η^1^;η^1^ coordinated mode. The dihedral angles of C_12_H_8_N_2_ with respect to the furan rings of the carboxylates that are coordinated to the same Cu atom are 62.18 (11)° and 88.27 (12)° (Fig. 1).

The Cu atom is linked by two furan-2,5-dicarboxylates to give rise to an infinite chain (Fig.2).

### Experimental

Furan-2,5-dicarboxylic acid (0.0156 g, 0.10 mmol), Cu(NO_3_)_2_^.^6H_2_O (0.0298 g, 0.10 mmol), and C_12_H_8_N_2_ (0.0198, 0.11 mmol) were dissolved in DMI (5 ml, 48 mmol) under stirring. The mixture with molar ratio of 1 furan-2,5-dicarboxylic acid: 1 Cu(NO_3_)_2_^.^6H_2_O: 1.1 C_12_H_8_N_2_: 480 DMI was heated at 393 K for 2 days. Blue block were collected as a single phase.

### Refinement

The carbon H-atoms were placed in calculated positions (C—H (furan ring and phen ring) = 0.93 Å, C—H (CH_2_) = 0.97 Å, C—H (CH_3_) = 0.98 Å) and were included in the refinement in the riding-model approximation, with *U*_iso_(H) = 1.2Ueq(C).

### Crystal data

**Table:**

[Cu(C_6_H_2_O_5_)(C_12_H_8_N_2_)(C_5_H_10_N_2_O)]	*F*(000) = 2104
*M**_r_* = 511.98	*D*_x_ = 1.602 Mg m^−^^3^
Orthorhombic, *P**b**c**a*	Mo *K*α radiation, λ = 0.71073 Å
Hall symbol: -P 2ac 2ab	Cell parameters from 2000 reflections
*a* = 15.620 (3) Å	θ = 3.0–25.0°
*b* = 14.598 (3) Å	µ = 1.08 mm^−^^1^
*c* = 18.616 (4) Å	*T* = 293 K
*V* = 4244.8 (15) Å^3^	Block, blue
*Z* = 8	0.10 × 0.10 × 0.10 mm

### Data collection

**Table:**

Rigaku R-AXIS RAPID diffractometer	3727 independent reflections
Radiation source: fine-focus sealed tube	2376 reflections with *I* > 2σ(*I*)
Graphite monochromator	*R*_int_ = 0.110
Detector resolution: 10.00 pixels mm^-1^	θ_max_ = 25.0°, θ_min_ = 3.0°
ω scans	*h* = −18→18
Absorption correction: multi-scan (*ABSCOR*; Higashi, 1995)	*k* = −17→17
*T*_min_ = 0.900, *T*_max_ = 0.900	*l* = −22→20
30193 measured reflections	

### Refinement

**Table:**

Refinement on *F*^2^	Primary atom site location: structure-invariant direct methods
Least-squares matrix: full	Secondary atom site location: difference Fourier map
*R*[*F*^2^ > 2σ(*F*^2^)] = 0.047	Hydrogen site location: inferred from neighbouring sites
*wR*(*F*^2^) = 0.109	H-atom parameters constrained
*S* = 1.04	*w* = 1/[σ^2^(*F*_o_^2^) + (0.0447*P*)^2^ + 0.2947*P*] where *P* = (*F*_o_^2^ + 2*F*_c_^2^)/3
3727 reflections	(Δ/σ)_max_ < 0.001
309 parameters	Δρ_max_ = 0.46 e Å^−^^3^
0 restraints	Δρ_min_ = −0.37 e Å^−^^3^

### Special details

**Table:**

Geometry. All e.s.d.'s (except the e.s.d. in the dihedral angle between two l.s. planes) are estimated using the full covariance matrix. The cell e.s.d.'s are taken into account individually in the estimation of e.s.d.'s in distances, angles and torsion angles; correlations between e.s.d.'s in cell parameters are only used when they are defined by crystal symmetry. An approximate (isotropic) treatment of cell e.s.d.'s is used for estimating e.s.d.'s involving l.s. planes.
Refinement. Refinement of *F*^2^ against ALL reflections. The weighted *R*-factor *wR* and goodness of fit *S* are based on *F*^2^, conventional *R*-factors *R* are based on *F*, with *F* set to zero for negative *F*^2^. The threshold expression of *F*^2^ > σ(*F*^2^) is used only for calculating *R*-factors(gt) *etc*. and is not relevant to the choice of reflections for refinement. *R*-factors based on *F*^2^ are statistically about twice as large as those based on *F*, and *R*- factors based on ALL data will be even larger.

### Fractional atomic coordinates and isotropic or equivalent isotropic displacement parameters (Å^2^)

**Table:**

	*x*	*y*	*z*	*U*_iso_*/*U*_eq_	
Cu1	0.24404 (3)	0.39844 (3)	0.43722 (2)	0.03211 (16)	
O1	0.14532 (17)	0.4367 (2)	0.38161 (13)	0.0416 (7)	
O2	0.12024 (19)	0.2971 (2)	0.33748 (15)	0.0486 (8)	
O3	−0.00981 (17)	0.36718 (18)	0.25302 (14)	0.0359 (6)	
O4	−0.17851 (18)	0.4375 (2)	0.13743 (13)	0.0427 (7)	
O5	−0.12344 (19)	0.2972 (2)	0.15283 (14)	0.0457 (8)	
O6	0.36449 (19)	0.5888 (2)	0.54050 (16)	0.0542 (8)	
N1	0.3387 (2)	0.3743 (2)	0.50900 (16)	0.0323 (8)	
N2	0.1712 (2)	0.3470 (2)	0.51765 (16)	0.0331 (8)	
N3	0.2429 (2)	0.5747 (3)	0.60884 (18)	0.0493 (9)	
N4	0.2290 (2)	0.5799 (2)	0.48911 (17)	0.0411 (9)	
C1	0.1065 (2)	0.3789 (3)	0.34036 (19)	0.0331 (9)	
C2	0.0400 (2)	0.4234 (3)	0.29553 (19)	0.0355 (10)	
C3	0.0156 (3)	0.5122 (3)	0.2869 (2)	0.0438 (11)	
H3	0.0392	0.5632	0.3094	0.053*	
C4	−0.0534 (3)	0.5117 (3)	0.2366 (2)	0.0434 (11)	
H4	−0.0833	0.5626	0.2200	0.052*	
C5	−0.0672 (3)	0.4228 (3)	0.21737 (19)	0.0355 (10)	
C6	−0.1268 (2)	0.3795 (3)	0.16585 (19)	0.0337 (10)	
C7	0.4221 (3)	0.3874 (3)	0.5029 (2)	0.0430 (10)	
H7	0.4432	0.4125	0.4605	0.052*	
C8	0.4805 (3)	0.3649 (3)	0.5581 (2)	0.0500 (12)	
H8	0.5391	0.3719	0.5511	0.060*	
C9	0.4493 (3)	0.3325 (3)	0.6221 (2)	0.0471 (12)	
H9	0.4868	0.3178	0.6591	0.057*	
C10	0.3614 (3)	0.3216 (3)	0.6314 (2)	0.0386 (10)	
C11	0.3216 (3)	0.2927 (3)	0.6978 (2)	0.0478 (11)	
H11	0.3558	0.2809	0.7377	0.057*	
C12	0.2360 (3)	0.2825 (3)	0.7033 (2)	0.0481 (12)	
H12	0.2123	0.2652	0.7470	0.058*	
C13	0.1811 (3)	0.2980 (3)	0.6430 (2)	0.0394 (10)	
C14	0.0921 (3)	0.2848 (3)	0.6432 (2)	0.0459 (11)	
H14	0.0648	0.2653	0.6848	0.055*	
C15	0.0454 (3)	0.3007 (3)	0.5821 (2)	0.0486 (12)	
H15	−0.0134	0.2907	0.5816	0.058*	
C16	0.0877 (3)	0.3324 (3)	0.5202 (2)	0.0417 (11)	
H16	0.0554	0.3436	0.4792	0.050*	
C17	0.2179 (3)	0.3287 (2)	0.5782 (2)	0.0324 (9)	
C18	0.3083 (3)	0.3418 (3)	0.57286 (19)	0.0323 (9)	
C19	0.2874 (3)	0.5825 (3)	0.5464 (2)	0.0400 (10)	
C20	0.1433 (3)	0.5936 (3)	0.5180 (2)	0.0526 (12)	
H20A	0.1018	0.5556	0.4932	0.063*	
H20B	0.1260	0.6572	0.5142	0.063*	
C21	0.1525 (3)	0.5647 (3)	0.5963 (3)	0.0528 (12)	
H21A	0.1196	0.6044	0.6277	0.063*	
H21B	0.1343	0.5019	0.6033	0.063*	
C22	0.2507 (3)	0.6290 (3)	0.4228 (2)	0.0554 (12)	
H22A	0.2415	0.6934	0.4296	0.066*	
H22B	0.2152	0.6073	0.3843	0.066*	
H22C	0.3098	0.6183	0.4112	0.066*	
C23	0.2837 (3)	0.5516 (3)	0.6760 (2)	0.0618 (15)	
H23A	0.2734	0.4883	0.6869	0.074*	
H23B	0.2607	0.5892	0.7136	0.074*	
H23C	0.3442	0.5621	0.6721	0.074*	

### Atomic displacement parameters (Å^2^)

**Table:**

	*U*^11^	*U*^22^	*U*^33^	*U*^12^	*U*^13^	*U*^23^
Cu1	0.0273 (3)	0.0421 (3)	0.0270 (2)	0.0001 (2)	0.0005 (2)	0.0016 (2)
O1	0.0385 (17)	0.0466 (17)	0.0397 (15)	−0.0005 (14)	−0.0093 (13)	0.0014 (14)
O2	0.048 (2)	0.049 (2)	0.0492 (19)	0.0075 (16)	−0.0027 (14)	−0.0014 (15)
O3	0.0321 (16)	0.0415 (15)	0.0342 (14)	0.0000 (13)	−0.0061 (12)	−0.0016 (14)
O4	0.0398 (17)	0.0527 (19)	0.0358 (15)	0.0101 (14)	−0.0112 (13)	−0.0074 (14)
O5	0.054 (2)	0.0447 (19)	0.0387 (16)	−0.0043 (15)	−0.0034 (14)	0.0004 (14)
O6	0.0372 (19)	0.064 (2)	0.062 (2)	−0.0037 (16)	0.0009 (15)	−0.0073 (16)
N1	0.0288 (19)	0.0338 (19)	0.0345 (18)	0.0003 (14)	0.0025 (14)	−0.0018 (15)
N2	0.031 (2)	0.037 (2)	0.0317 (18)	−0.0033 (15)	−0.0017 (15)	−0.0043 (15)
N3	0.047 (2)	0.057 (2)	0.0432 (19)	−0.004 (2)	0.0001 (19)	−0.0054 (17)
N4	0.039 (2)	0.047 (2)	0.0373 (18)	−0.0009 (16)	−0.0022 (15)	−0.0010 (17)
C1	0.030 (2)	0.043 (3)	0.027 (2)	−0.0016 (19)	0.0059 (16)	−0.0017 (19)
C2	0.027 (2)	0.052 (3)	0.028 (2)	−0.0020 (19)	0.0009 (16)	−0.0091 (19)
C3	0.041 (3)	0.047 (3)	0.043 (2)	−0.001 (2)	−0.006 (2)	−0.004 (2)
C4	0.050 (3)	0.040 (3)	0.040 (2)	0.006 (2)	−0.008 (2)	0.002 (2)
C5	0.030 (2)	0.048 (3)	0.028 (2)	0.0077 (19)	0.0003 (17)	0.0025 (19)
C6	0.027 (2)	0.050 (3)	0.024 (2)	0.001 (2)	0.0043 (16)	0.0016 (19)
C7	0.033 (2)	0.055 (3)	0.041 (2)	−0.002 (2)	−0.0002 (18)	−0.004 (2)
C8	0.031 (2)	0.064 (3)	0.055 (3)	0.004 (2)	−0.003 (2)	−0.007 (3)
C9	0.048 (3)	0.049 (3)	0.045 (3)	0.009 (2)	−0.014 (2)	−0.004 (2)
C10	0.046 (3)	0.034 (2)	0.035 (2)	0.008 (2)	−0.005 (2)	−0.0051 (19)
C11	0.070 (3)	0.038 (3)	0.035 (2)	0.006 (2)	−0.006 (2)	0.002 (2)
C12	0.067 (4)	0.044 (3)	0.033 (2)	0.002 (2)	0.009 (2)	0.004 (2)
C13	0.056 (3)	0.029 (2)	0.033 (2)	−0.006 (2)	0.010 (2)	−0.0004 (18)
C14	0.053 (3)	0.036 (2)	0.049 (3)	−0.008 (2)	0.024 (2)	−0.002 (2)
C15	0.038 (3)	0.049 (3)	0.059 (3)	−0.009 (2)	0.015 (2)	−0.010 (2)
C16	0.034 (3)	0.050 (3)	0.041 (2)	−0.004 (2)	0.0025 (19)	−0.003 (2)
C17	0.038 (2)	0.023 (2)	0.036 (2)	−0.0011 (16)	0.0038 (17)	−0.0009 (17)
C18	0.036 (2)	0.031 (2)	0.030 (2)	0.0029 (17)	−0.0019 (18)	−0.0036 (18)
C19	0.043 (3)	0.028 (2)	0.048 (3)	0.0004 (18)	0.003 (2)	−0.0043 (19)
C20	0.039 (3)	0.052 (3)	0.067 (3)	0.009 (2)	−0.002 (2)	−0.016 (2)
C21	0.049 (3)	0.045 (3)	0.064 (3)	−0.004 (2)	0.013 (2)	−0.009 (2)
C22	0.065 (3)	0.048 (3)	0.053 (3)	−0.004 (3)	0.000 (3)	0.003 (2)
C23	0.088 (4)	0.051 (3)	0.046 (3)	0.004 (3)	−0.007 (3)	−0.005 (2)

### Geometric parameters (Å, º)

**Table:**

Cu1—O4^i^	1.929 (3)	C7—H7	0.9300
Cu1—O1	1.939 (3)	C8—C9	1.371 (6)
Cu1—N1	2.024 (3)	C8—H8	0.9300
Cu1—N2	2.025 (3)	C9—C10	1.394 (6)
Cu1—N4	2.829 (3)	C9—H9	0.9300
O1—C1	1.292 (5)	C10—C18	1.400 (5)
O2—C1	1.214 (5)	C10—C11	1.448 (6)
O3—C5	1.380 (4)	C11—C12	1.349 (6)
O3—C2	1.380 (5)	C11—H11	0.9300
O4—C6	1.284 (5)	C12—C13	1.431 (6)
O4—Cu1^ii^	1.929 (3)	C12—H12	0.9300
O5—C6	1.228 (5)	C13—C14	1.402 (6)
O6—C19	1.213 (5)	C13—C17	1.410 (5)
N1—C7	1.321 (5)	C14—C15	1.371 (6)
N1—C18	1.366 (5)	C14—H14	0.9300
N2—C16	1.323 (5)	C15—C16	1.405 (6)
N2—C17	1.369 (5)	C15—H15	0.9300
N3—C19	1.360 (5)	C16—H16	0.9300
N3—C21	1.438 (5)	C17—C18	1.428 (6)
N3—C23	1.443 (5)	C20—C21	1.524 (6)
N4—C19	1.403 (5)	C20—H20A	0.9700
N4—C20	1.457 (5)	C20—H20B	0.9700
N4—C22	1.467 (5)	C21—H21A	0.9700
C1—C2	1.483 (5)	C21—H21B	0.9700
C2—C3	1.360 (6)	C22—H22A	0.9600
C3—C4	1.428 (5)	C22—H22B	0.9600
C3—H3	0.9300	C22—H22C	0.9600
C4—C5	1.363 (6)	C23—H23A	0.9600
C4—H4	0.9300	C23—H23B	0.9600
C5—C6	1.479 (5)	C23—H23C	0.9600
C7—C8	1.412 (6)		
			
O4^i^—Cu1—O1	91.66 (12)	C9—C10—C18	117.6 (4)
O4^i^—Cu1—N1	93.94 (12)	C9—C10—C11	124.2 (4)
O1—Cu1—N1	169.56 (12)	C18—C10—C11	118.1 (4)
O4^i^—Cu1—N2	174.20 (13)	C12—C11—C10	121.5 (4)
O1—Cu1—N2	93.14 (12)	C12—C11—H11	119.3
N1—Cu1—N2	81.83 (13)	C10—C11—H11	119.3
O4^i^—Cu1—N4	91.21 (11)	C11—C12—C13	121.2 (4)
O1—Cu1—N4	81.19 (11)	C11—C12—H12	119.4
N1—Cu1—N4	89.88 (11)	C13—C12—H12	119.4
N2—Cu1—N4	92.75 (11)	C14—C13—C17	116.7 (4)
C1—O1—Cu1	120.1 (3)	C14—C13—C12	124.8 (4)
C5—O3—C2	107.0 (3)	C17—C13—C12	118.5 (4)
C6—O4—Cu1^ii^	119.8 (3)	C15—C14—C13	120.1 (4)
C7—N1—C18	117.9 (3)	C15—C14—H14	119.9
C7—N1—Cu1	129.6 (3)	C13—C14—H14	119.9
C18—N1—Cu1	112.4 (3)	C14—C15—C16	119.1 (4)
C16—N2—C17	117.7 (4)	C14—C15—H15	120.5
C16—N2—Cu1	129.9 (3)	C16—C15—H15	120.5
C17—N2—Cu1	112.4 (3)	N2—C16—C15	123.1 (4)
C19—N3—C21	111.8 (4)	N2—C16—H16	118.5
C19—N3—C23	122.3 (4)	C15—C16—H16	118.5
C21—N3—C23	123.4 (4)	N2—C17—C13	123.3 (4)
C19—N4—C20	108.2 (3)	N2—C17—C18	116.4 (4)
C19—N4—C22	118.4 (4)	C13—C17—C18	120.4 (4)
C20—N4—C22	117.1 (4)	N1—C18—C10	123.0 (4)
C19—N4—Cu1	103.4 (2)	N1—C18—C17	116.8 (3)
C20—N4—Cu1	109.3 (3)	C10—C18—C17	120.2 (4)
C22—N4—Cu1	98.7 (2)	O6—C19—N3	126.3 (4)
O2—C1—O1	125.9 (4)	O6—C19—N4	125.4 (4)
O2—C1—C2	122.0 (4)	N3—C19—N4	108.4 (4)
O1—C1—C2	112.1 (4)	N4—C20—C21	103.2 (4)
C3—C2—O3	109.9 (4)	N4—C20—H20A	111.1
C3—C2—C1	132.9 (4)	C21—C20—H20A	111.1
O3—C2—C1	117.2 (4)	N4—C20—H20B	111.1
C2—C3—C4	106.5 (4)	C21—C20—H20B	111.1
C2—C3—H3	126.7	H20A—C20—H20B	109.1
C4—C3—H3	126.7	N3—C21—C20	102.7 (4)
C5—C4—C3	107.2 (4)	N3—C21—H21A	111.2
C5—C4—H4	126.4	C20—C21—H21A	111.2
C3—C4—H4	126.4	N3—C21—H21B	111.2
C4—C5—O3	109.3 (3)	C20—C21—H21B	111.2
C4—C5—C6	132.6 (4)	H21A—C21—H21B	109.1
O3—C5—C6	118.0 (4)	N4—C22—H22A	109.5
O5—C6—O4	126.3 (4)	N4—C22—H22B	109.5
O5—C6—C5	121.3 (4)	H22A—C22—H22B	109.5
O4—C6—C5	112.4 (4)	N4—C22—H22C	109.5
N1—C7—C8	122.7 (4)	H22A—C22—H22C	109.5
N1—C7—H7	118.7	H22B—C22—H22C	109.5
C8—C7—H7	118.7	N3—C23—H23A	109.5
C9—C8—C7	118.9 (4)	N3—C23—H23B	109.5
C9—C8—H8	120.5	H23A—C23—H23B	109.5
C7—C8—H8	120.5	N3—C23—H23C	109.5
C8—C9—C10	119.8 (4)	H23A—C23—H23C	109.5
C8—C9—H9	120.1	H23B—C23—H23C	109.5
C10—C9—H9	120.1		
			
O4^i^—Cu1—O1—C1	93.0 (3)	Cu1—N1—C7—C8	−178.5 (3)
N1—Cu1—O1—C1	−144.6 (6)	N1—C7—C8—C9	−3.5 (7)
N2—Cu1—O1—C1	−83.8 (3)	C7—C8—C9—C10	0.6 (7)
N4—Cu1—O1—C1	−176.1 (3)	C8—C9—C10—C18	2.2 (6)
O4^i^—Cu1—N1—C7	2.9 (4)	C8—C9—C10—C11	−176.6 (4)
O1—Cu1—N1—C7	−119.4 (7)	C9—C10—C11—C12	−179.3 (4)
N2—Cu1—N1—C7	178.9 (4)	C18—C10—C11—C12	2.0 (6)
N4—Cu1—N1—C7	−88.3 (4)	C10—C11—C12—C13	1.4 (7)
O4^i^—Cu1—N1—C18	−178.8 (3)	C11—C12—C13—C14	176.9 (4)
O1—Cu1—N1—C18	58.9 (8)	C11—C12—C13—C17	−3.1 (6)
N2—Cu1—N1—C18	−2.8 (3)	C17—C13—C14—C15	0.7 (6)
N4—Cu1—N1—C18	90.0 (3)	C12—C13—C14—C15	−179.3 (4)
O1—Cu1—N2—C16	10.7 (4)	C13—C14—C15—C16	−1.6 (6)
N1—Cu1—N2—C16	−178.5 (4)	C17—N2—C16—C15	1.1 (6)
N4—Cu1—N2—C16	92.0 (4)	Cu1—N2—C16—C15	−176.3 (3)
O1—Cu1—N2—C17	−166.8 (3)	C14—C15—C16—N2	0.7 (7)
N1—Cu1—N2—C17	4.0 (3)	C16—N2—C17—C13	−2.0 (6)
N4—Cu1—N2—C17	−85.5 (3)	Cu1—N2—C17—C13	175.8 (3)
O4^i^—Cu1—N4—C19	−97.5 (3)	C16—N2—C17—C18	177.6 (4)
O1—Cu1—N4—C19	171.0 (3)	Cu1—N2—C17—C18	−4.5 (4)
N1—Cu1—N4—C19	−3.6 (3)	C14—C13—C17—N2	1.1 (6)
N2—Cu1—N4—C19	78.3 (3)	C12—C13—C17—N2	−178.9 (3)
O4^i^—Cu1—N4—C20	147.4 (3)	C14—C13—C17—C18	−178.5 (4)
O1—Cu1—N4—C20	55.9 (3)	C12—C13—C17—C18	1.5 (6)
N1—Cu1—N4—C20	−118.6 (3)	C7—N1—C18—C10	−0.2 (6)
N2—Cu1—N4—C20	−36.8 (3)	Cu1—N1—C18—C10	−178.8 (3)
O4^i^—Cu1—N4—C22	24.6 (3)	C7—N1—C18—C17	179.7 (4)
O1—Cu1—N4—C22	−66.9 (3)	Cu1—N1—C18—C17	1.2 (4)
N1—Cu1—N4—C22	118.5 (3)	C9—C10—C18—N1	−2.4 (6)
N2—Cu1—N4—C22	−159.7 (3)	C11—C10—C18—N1	176.4 (4)
Cu1—O1—C1—O2	8.0 (5)	C9—C10—C18—C17	177.6 (4)
Cu1—O1—C1—C2	−172.7 (2)	C11—C10—C18—C17	−3.6 (6)
C5—O3—C2—C3	−0.4 (4)	N2—C17—C18—N1	2.3 (5)
C5—O3—C2—C1	179.2 (3)	C13—C17—C18—N1	−178.1 (3)
O2—C1—C2—C3	−176.7 (4)	N2—C17—C18—C10	−177.8 (3)
O1—C1—C2—C3	4.0 (6)	C13—C17—C18—C10	1.9 (6)
O2—C1—C2—O3	3.8 (5)	C21—N3—C19—O6	−177.5 (4)
O1—C1—C2—O3	−175.5 (3)	C23—N3—C19—O6	−14.8 (7)
O3—C2—C3—C4	0.2 (5)	C21—N3—C19—N4	1.1 (5)
C1—C2—C3—C4	−179.3 (4)	C23—N3—C19—N4	163.8 (4)
C2—C3—C4—C5	0.0 (5)	C20—N4—C19—O6	−166.9 (4)
C3—C4—C5—O3	−0.3 (5)	C22—N4—C19—O6	−30.6 (6)
C3—C4—C5—C6	−177.4 (4)	Cu1—N4—C19—O6	77.2 (4)
C2—O3—C5—C4	0.4 (4)	C20—N4—C19—N3	14.4 (4)
C2—O3—C5—C6	178.0 (3)	C22—N4—C19—N3	150.8 (4)
Cu1^ii^—O4—C6—O5	−3.8 (5)	Cu1—N4—C19—N3	−101.4 (3)
Cu1^ii^—O4—C6—C5	175.0 (2)	C19—N4—C20—C21	−22.9 (4)
C4—C5—C6—O5	174.0 (4)	C22—N4—C20—C21	−159.9 (4)
O3—C5—C6—O5	−2.8 (5)	Cu1—N4—C20—C21	89.0 (3)
C4—C5—C6—O4	−4.8 (6)	C19—N3—C21—C20	−15.0 (5)
O3—C5—C6—O4	178.3 (3)	C23—N3—C21—C20	−177.5 (4)
C18—N1—C7—C8	3.2 (6)	N4—C20—C21—N3	22.3 (4)

Symmetry codes: (i) *x*+1/2, *y*, −*z*+1/2; (ii) *x*−1/2, *y*, −*z*+1/2.
